# Predicting gene disease associations with knowledge graph embeddings for diseases with curtailed information

**DOI:** 10.1093/nargab/lqae049

**Published:** 2024-05-14

**Authors:** Francesco Gualdi, Baldomero Oliva, Janet Piñero

**Affiliations:** Integrative Biomedical Informatics, Research Programme on Biomedical Informatics (IBI-GRIB), Hospital del Mar Medical Research Institute (IMIM), Department of Experimental and Health Sciences, Universitat Pompeu Fabra, C/Dr Aiguader 88, E-08003 Barcelona, Spain; Structural Bioinformatics Lab, Research Programme on Biomedical Informatics (SBI-GRIB), Department of Experimental and Health Sciences, Universitat Pompeu Fabra, C/Dr Aiguader 88, E-08003 Barcelona, Spain; Structural Bioinformatics Lab, Research Programme on Biomedical Informatics (SBI-GRIB), Department of Experimental and Health Sciences, Universitat Pompeu Fabra, C/Dr Aiguader 88, E-08003 Barcelona, Spain; Integrative Biomedical Informatics, Research Programme on Biomedical Informatics (IBI-GRIB), Hospital del Mar Medical Research Institute (IMIM), Department of Experimental and Health Sciences, Universitat Pompeu Fabra, C/Dr Aiguader 88, E-08003 Barcelona, Spain; Medbioinformatics Solutions SL, Barcelona, Spain

## Abstract

Knowledge graph embeddings (KGE) are a powerful technique used in the biomedical domain to represent biological knowledge in a low dimensional space. However, a deep understanding of these methods is still missing, and, in particular, regarding their applications to prioritize genes associated with complex diseases with reduced genetic information. In this contribution, we built a knowledge graph (KG) by integrating heterogeneous biomedical data and generated KGE by implementing state-of-the-art methods, and two novel algorithms: Dlemb and BioKG2vec. Extensive testing of the embeddings with unsupervised clustering and supervised methods showed that KGE can be successfully implemented to predict genes associated with diseases and that our novel approaches outperform most existing algorithms in both scenarios. Our findings underscore the significance of data quality, preprocessing, and integration in achieving accurate predictions. Additionally, we applied KGE to predict genes linked to Intervertebral Disc Degeneration (IDD) and illustrated that functions pertinent to the disease are enriched within the prioritized gene set.

## Graphical Abstract

**Graphical Abstract:**
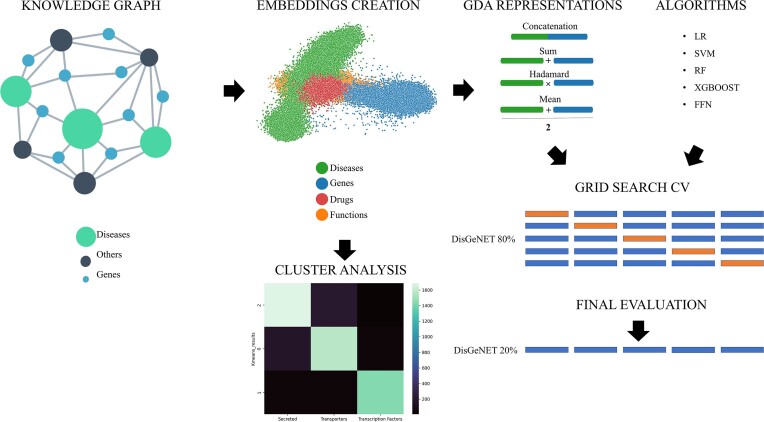

## Introduction

Predicting genes associated with diseases is a challenging task. Recent advancements in genomic technologies have contributed to reach a deeper understanding of the genetics underlying complex diseases. However, the difficulties related to costs and time of these technologies have prompted the development of *in silico* methods to perform this task (1).

In this regard, network approaches have emerged as valuable tools for building meaningful models allowing the integration of heterogeneous biological knowledge from numerous sources (2). These heterogeneous networks, defined as knowledge graphs (KGs), contain structured depictions of biological systems wherein different biological entities interact through complex relationships. Elucidating these intricate relations is crucial to better interpret complex biological data and thus the plausible causes of diseases.

KGs are increasingly implemented in the biomedical field due to their potential in representing and analyzing complex biomedical data. Recent research has highlighted their importance in enabling intelligent applications such as recommendation systems, semantic search, and logical reasoning. Automated schemes have been shown to significantly reduce the cost of building knowledge graphs (3). Current research is addressing challenges such as knowledge graph completion and extraction methods for unstructured data. There is also a growing emphasis on constructing KGs from natural language text, with a focus on named entity recognition and relation extraction (4). The field is still facing technical challenges, but the ongoing research aims to enhance the quality and reliability of knowledge graphs through novel techniques, models, and frameworks.

One commonly used approach to infer new interactions between biological entities involves expressing the entities within KGs as low-dimensional vectors using vectorial representations that preserve the graph's local structure known as knowledge graph embeddings (KGE). This method outperforms other approaches in terms of accuracy and scalability of their prediction (5).

Numerous methods have been developed to generate embeddings from KGs, and they can be broadly categorized into five main families: translational models, matrix factorization, semantic matching, random walks-based models, and deep neural networks. Refer to (6,7) for a comprehensive overview of these methods. Recently, new techniques that combine these existing methods have emerged (8). For example translational methods or PageRank (9) are merged with graph attention networks (GAT) to improve predictive powers of the embeddings (10). Several studies have been conducted to explore the potential of KGE for predicting gene-disease associations (GDAs). For instance, Nunes *et al.* investigated the impact of employing rich semantic representations based on more than one ontology to predict GDAs by testing different embedding creation models and machine learning algorithms (11). Other works have focused on the heterogeneous integration of knowledge bases with the development of a single deep learning framework for predicting GDAs starting from a KG (12,13).

In the biomedical domain, KGE have been implemented for a wide range of downstream machine learning tasks, such as the prediction of drug – target (14) and protein–protein interactions (15), as well as therapeutic indications (16). Also, KGE have demonstrated the ability to achieve prediction capabilities similar to those of raw data, while also offering the advantage of reduced dimensionality compared to the original dataset (17). While previous studies have made progress in implementing KGE methods in GDA research, we lack a proper benchmark of available methods. Existing works in this field are limited to evaluating the proposed method (12) or the comparison of different algorithms (11) without providing a deeper insight into the generated embeddings or validating a particular use case. In this work we conducted a comparison of different methods of KGE creation with unsupervised and supervised machine learning tasks. We first generated KGE from multiple ontologies and biological knowledge bases, and we implemented four state-of-the-art methods, and two novel algorithms. Subsequently, we analyzed the generated embeddings using unsupervised clustering algorithms. Furthermore, we evaluated the performance of the embeddings in a GDAs prediction task. Finally, we used the best performing model to predict potential genes associated to intervertebral disc degeneration (IDD).

## Materials and methods

### Data sources

To build the KG, we mined different types of biological data from publicly available repositories:

*Protein–protein interactions:* We partially integrated data from multiscale interactome (downloaded 29/06/2022) (16). Specifically, the data were integrated from:

- The biological general repository for interaction dataset (BioGRID) (18). This is a repository of manually curated both physical and genetic interactions between proteins from 71 713 high—throughput and low—throughput publications.
- The database of interacting proteins (DIP) (19) in which only physical protein - protein interactions are reported with experimental and curated evidence.
- Four protein-protein interaction networks from the human reference protein interactome mapping project (20)): (HI-I-05: 2611 interactions between 1522 proteins; HI-II-14 13 426 interactions between 4228 proteins, Venkatesan-09: 233 interactions between 229 proteins; Yu-11 1126 interactions between 1126 proteins). In addition, we integrated the last version of the Human reference interactome (HI-III-20) (20).
- Physical protein-protein interaction from Menche *et al.* (21)). This repository integrates different resources of physical protein - protein interaction data from experimental evidence. It integrates regulatory interactions from TRANSFAC (22) database, binary interactions from yeast-two-hybrid datasets and curated interactions from IntAct (23), BioGRID and HPRD (24). It integrates also metabolic-enzyme interactions from KEGG (25) and BIGG (26), protein complex interactions from CORUM (27), kinase-substrate interactions from PhosphositePlus (28) and signalling interactions from Vinayagam *et al.* (29)

Only human proteins for which existed direct experimental evidence of a physical interaction were considered.

*Ontologies:* Ontologies are computational structures that aim to describe and classify the entities belonging to a certain domain in a structured and machine-readable format in order to be implemented in a broad range of applications. The main components of the ontology are classes that represent specific entities and usually are associated with an identifier. These classes are arranged in a hierarchical way from general to more specific and are connected to each other through relations. Finally, ontologies feature metadata, formats and axioms (30) For our purpose we integrated the following types of ontologies:

- Gene Ontology (GO) (31), (downloaded 18/07/2022) is a knowledge base that aims to computationally describe biological systems ranging from molecules to organisms, as of 2023 it comprises 43 248 terms, 7 503 460 annotations across 5267 species.
- Disease Ontology (DO) (32), (downloaded 02/08/2022) is an ontological structure of standardized disease descriptors across multiple resources. The aim of the project is to provide a computable structure of integrated biomedical data in order to improve the knowledge on human diseases.
- Human Phenotype Ontology (HPO) (33), (downloaded 22/08/2022) is a comprehensive logical structure that describes phenotypic abnormalities found in human diseases. This enables computational inference and interoperability in digital medicine.

We integrated HPO and DO and mapped the common codes to UMLS CUIS (34)

*Gene product annotations to biological processes:* Proteins in the KG were mapped to their specific biological process through GO. GO annotations are statements about the function of a particular gene product, in this way, it is possible to obtain a snapshot of the current biological knowledge. We included gene annotations from the gene ontology association file (downloaded 29/06/2022).

*Gene products annotations to phenotypes:* We integrated data of genes associated to phenotypes from two sources:

- DisGeNET (35) is one of the largest publicly available collections of genes and variants associated with human diseases, it integrates GDAs data from curated resources with data automatically mined from the scientific literature using text-mining approaches. For our purposes we exploited DisGeNET curated (version 7.0) that integrates expert curated human gene disease associations from different data sources. To create a dataset, we used curated data from DisGeNET, comprising a total of 84 037 associations (hereafter considered as positives). We generated the same number of gene-disease non-associations (i.e. negatives) by considering that such associations were not reported in the text—mining version of DisGeNET, hence taking randomly any gene-disease pair not reported as positive.
- HPO gene annotations to phenotypes: HPO (downloaded 02/08/2022) provides a file that links between genes and HPO terms. If variants in a specific gene are associated with a disease, then all the phenotypes related to that specific disease are assigned to that gene.

*Phenotypes annotated to diseases:* We integrated annotations of phenotypes to disease from the phenotype.hpoa file from HPO ontology (downloaded 15/12/2022).

*Drug-disease associations:* We integrated data of drug-disease pairs from the multiscale interactome (16). This dataset is integrated by a collection of FDA approved treatments for diseases including different sources:

- The drug repurposing database (36) is a database of gold-standard drug-disease pairs extracted from DrugCentral (37) and ClinicalTrials.gov
- The drug repurposing hub (38) is a collection of drug-disease including 4707 compounds. The database contains information mined from publicly and proprietary datasets that undergo manual curation.
- The drug indication database (39) integrates data from 12 openly available, commercially available and proprietary information sources.

The dataset was filtered by keeping only human proteins resulting in a total number of drug - disease pairs of 5926.

*Drug–target interaction* We obtained a dataset of drugs and their mode of actions on target proteins by integrating DrugBank (40) and the drug repurposing hub. Proteins that were not included in the protein – protein interaction network were removed.

### KGE generation algorithms

We tested four state-of-the-art algorithms based on different principles and we implemented two novel methods to generate embeddings, referred to as BioKG2vec and Dlemb. For all experiments, the embeddings vector dimension was 100 and we set the number of epochs to 15.

*RotatE:* RotatE (41) is a KGE generation algorithm that maps relations and entities to the complex vector space. The relations are considered as rotations from the source entity to the target entity. The principle lays on the assumption that given the triple ‘(*h*,*r*,*t*)’, where *h* is the head, *r* is the relation, *t* is the tail e.g. ‘(protein1, interacts with, protein2)’the embeddings are obtained by the relation *t* = *h* ◦ *r* where ◦ denotes the Hadamard operation between the *h* and *r* vectors.

*Relational graph convolutional networks (R-GCN)* (42): R-GCN is an architecture for calculating the forward pass of relational graphs with multiple edge types. The propagation model is calculated as follows:


\begin{equation*}h_i^{\left( {h + 1} \right)} = \sigma \left( {\mathop \sum \limits_{r\epsilon R} \mathop \sum \limits_{j\epsilon N_i^r} \frac{1}{{{{c}_{i,r}}}}W_r^{\left( l \right)}h_j^{\left( l \right)} + W_0^{\left( l \right)}h_i^{\left( l \right)}} \right)\end{equation*}

where $N_i^r$ is the set of neighbours of node *i* under the relation $r\ \in R$ and ${{c}_{i,r}}$ is a problem-specific normalization constant that is chosen beforehand. $h_j^{( l )}$ is the node vector of neighbour *j* on which applies weight matrix $W_r^{( l )}$ of relation r in the iteration *l*. $W_0^{( l )}h_i^{( l )}$ is the representation of node *i* at layer *l* i.e. a self-representation at antecedent iteration.

*Metapath2Vec* (43) is an extension of the Node2Vec model (44) well suited for heterogeneous networks. The algorithm relies on meta-path-based random walks that capture both semantic and structural correlations between different types of nodes.

*BioKG2vec:* BioKG2vec relies on a biased random-walk approach in which the user can prioritize specific connections by assigning a weight to edges. In the KG defined in this work we used four different node-types: drug, protein, function and disease. Then, the probability of visiting a specific neighbour at every step is given by the equation:


\begin{equation*}P\left( {{{n}_i}} \right) = \frac{{\left( {{{n}_i}\left( {1 + \frac{{{{w}_i}}}{{{{n}_i}}}} \right)} \right)}}{W}\end{equation*}

where $P( {{{n}_i}} )$ is the probability for the random walker to visit a specific node type, ${{n}_i}$ is the number of paths leading to the node (of the same type), ${{w}_i}$ is the assigned weight (also specific for the type) and $W$ equals to the node degree plus the sum of all weights (i.e. $\mathop \sum \limits_i {{w}_i}$)$.$ To detect the optimal weights for the prediction of GDAs we performed a grid search assigning weights prioritizing drug → protein → function → disease. Moreover, the walker stores the information of the visited edge type, and this information is used as input for Word2Vec algorithm in the embedding generation step. Thus, the algorithm handles different edges and nodes behaving differently for each node type being visited and storing the edge type of information too. BioKG2vec is available at https://zenodo.org/badge/latestdoi/624339823.

*Dlemb:* Dlemb is a shallow neural network (NN) that consists of three layers: the input layer, embedding layer and output layer. The input layer takes as input KG entities as numbers and outputs them to the embedding layer. In the dot layer the scalar product of the vector is computed and normalized so the result is a number that ranges between −1 and 1. A false relation yields −1 while true relations produce +1. Then, the RMSE is calculated between the dot product and the expected value. Finally, the ADAM optimizer is used to adjust the embeddings layer directly since these are parameters of the neural network so that the model can be fitted to the data.

Dlemb is available at https://zenodo.org/badge/latestdoi/635382680.

### Methods to combine embeddings

We used four strategies to combine gene and disease embeddings to obtain GDAs representations: (i) sum, which consisted of the addition of both vectors; (ii) average, in which we averaged them; (iii) concatenation, in which the result is a vector in a larger dimension, representing a pair gene–disease by concatenating both vectors; (iv) Hadamard product (i.e. each element is produced by the product of the elements of the two vectors). For this work, we produced embeddings of fixed dimension (i.e. 100) in the space of reals (i.e. ${{\mathbb{R}}^{100}}$).

### Unsupervised analysis of the embeddings

We assessed the quality of the embeddings performing k-means unsupervised clustering. Specifically, we used function and compartment-based classification to group gene products in 16 different categories from human protein atlas (HPA) (45). For diseases, we used annotations from UMLS to ICD-9 (46), that classify diseases into macro classes. We then used various evaluation scores for the comparison, such as the silhouette score, defined as:


\begin{equation*}\frac{{b\ - \ a}}{{max\left( {a,\ b} \right)}}\end{equation*}

where $b$ is the mean distance between a sample and all other points in the nearest cluster (nearest – cluster distance) and $a$ is the mean distance between a sample and all other points in the same class (inter – cluster distance). We calculated this score for different cluster sizes ranging from 10 to 20 for genes (the gold standard number of clusters is 16) and from 10 to 20 for diseases (the gold standard number of clusters is 16).

Finally, we evaluate the homogeneity score, defined as:


\begin{equation*}1{\mathrm{\ }} - {\mathrm{\ }}\frac{{H\left( {{{Y}_{true}}|{{Y}_{pred}}} \right)}}{{H\left( {{{Y}_{true}}} \right)}}\end{equation*}

That is a measure that quantifies the similarity of samples in each cluster. Where the ${{Y}_{true}}$ is the number of classes, ${{Y}_{pred}}$ is the number of clusters and $H({{Y}_{true}}|{{Y}_{pred}})$ represents the ratio between the number of classes ${{Y}_{true}}$ in cluster ${{Y}_{pred}}$ and the total number of samples in cluster ${{Y}_{pred}}$. When all the entities in the cluster belong to a class the homogeneity score equals 1.

Then, for visualization purposes, we performed UMAP dimensionality reduction on the embeddings and plotted the first two UMAP embeddings of gene and disease embeddings. Only three classes of genes and diseases are plotted.

#### Grid search to select the best predictive model

We performed a grid search cross-validation to find the best combination of embedding creation algorithm, GDAs representation and predictive machine learning (ML) and deep learning (DL) algorithms implemented in Scikit-learn (47) and Pytorch (48) respectively. In the grid-search experiment we created a KG in which we integrated all the biological data and 80% of curated GDAs from DisGeNET. We tested the predictions in the remaining 20% of GDAs that were not used in the embeddings creation step. To avoid data leakage, we excluded diseases with over 20 associated genes, of which >90% were shared with another disease. Additionally, we made sure that in the validation dataset there were no GDAs included in the HPO data. For each algorithm, we fitted a grid of parameters (Table 1) maximizing the area under the receiver operating-characteristic curve (ROCAUC). With this, we tested a total of 120 combinations for the grid search (Supplementary Table S1). Then, the best parameter combination was evaluated on the test set by assessing additional evaluation metrics, such as:


\begin{equation*}accuracy = \frac{{TP + TN}}{{TP + FP + TN + FN}}\end{equation*}

\begin{equation*}recall = \frac{{TP}}{{TP + FN}}\end{equation*}

\begin{equation*}precision = \frac{{TP}}{{TP + FP}}\end{equation*}

\begin{equation*}F1 = 2 \times \frac{{precision \times recall}}{{precision + recall}}\end{equation*}

\begin{equation*}FPR = \frac{{FP}}{{FP + TN}}\end{equation*}

**Table 1.** Search spaces of the algorithms tested during the grid search cross validation

Algorithm	Parameters	Values
LR	*C*	0.001, 0.01, 1, 5, 10, 25
	Penalty	L1, L2
Random Forest	Max depth	2, 4, 6, none
	No. of estimators	20, 50, 100
XGBOOST	Cosample by tree	0.3, 0.7
	Gamma	0, 0.5
	Learning rate	0.03, 0.3
	Max depth	2, 6
	No. of estimators	100, 150
	Subsample	0.4, 0.6
SVM	*C*	0.1, 1, 10
	Gamma	0.001, 0.01, 0.1
	Kernel	rbf, poly
FFN	No. of layers	2, 3
	No. of nodes first layer	50, 100, 150
	No. of nodes second layer	20, 50
	Activation function	sigmoid, tanh, relu
	Loss function	Binary cross-entropy, hinge
	Batch size	30, 100
	Epochs	20, 60

We also report the area under the precision recall curve (AUPRC).

### Ontology preprocessing and heterogeneous data integration

Once we selected the model with the highest predictive power, we investigated the influence of integrating heterogeneous biological data in the KG on the GDAs predictions. For this experiment we only used ontological data. Ontologies are complex, standardized data structures composed of classes, relations, axioms and metadata all of which are included in the raw ontology. Moreover, we tested the effect of implementing a pre-processing step in the ontology in which only classes and relations were maintained as a graph structure (axioms and metadata were excluded). We studied the following combinations of data sources:

1. HPO + HPO annotations raw
2. HPO + HPO annotations pre-processed
3. HPO + HPO annotations + GO + GO annotations (all) pre-processed

We used a comparison based on two metrics. For this experiment, we created embeddings with the Metapath2Vec algorithm, using concatenation for GDAs representation, and SVM as classification algorithm. For the processing of the ontologies nxontology and pronto (49) python libraries were used.

### Influence of GDAs in the KG for GDA-predictions

We tested the influence of adding increasing GDAs proportions in the KG. For this experiment, we used 20%, 50%, 80% and 100% of DisGeNET and we included it in the KG. Then we generated embeddings from the KGs with Metapath2Vec and we trained a SVM on 80% of DisGeNET. We tested the model on the 20% of remaining associations and calculated ROCAUC and AUPRC as evaluation metrics.

### Comparison with randomly generated embeddings

To show that the information is efficiently translated from the KG to the vectorial space, we compared the performance of Metapath2Vec generated embeddings and random embeddings of the same size. We aimed to assess the effectiveness of translating the information encoded in the KG into embeddings by comparing KGE with a null model. To conduct this evaluation, we created 100-dimensional random embeddings for each gene and disease, represented GDAs through concatenation, and tested their predictive capabilities. The number of associations is a latent variable that can be learned by ML to produce good predictions. This can be considered a potential bias. Therefore, we further tested the effect of removing the number of associations stratifying DisGeNET diseases by the number of associated genes. We divided the data into 23 groups in which the number of associations for every disease has a maximum difference of 20. Then we selected a disease belonging to every class, generated negative associations and performed a five-fold cross validation on the data with the best performing algorithm. We evaluated accuracy, precision, recall, F1 score and ROCAUC across every fold.

### Generalizability of the model

The predictive model selected was tested to predict associations for diseases not used in the training set. The rationale behind this experiment was to understand the capabilities of the model to predict gene-disease associations of new diseases, proving that the biological information encoded in the embeddings was generalizable.

To assess this, we trained the model on GDAs belonging to diseases of a specific ICD-9 disease class and then we tested the model on all other classes.

### Performance of the algorithms

We compared the performance of the algorithms with the top predictive power i.e. Metapath2Vec, BioKG2vec and Dlemb. We performed *n* = 10 experiments by randomly selecting 1000 nodes from the knowledge graph, creating the subnetwork and producing the embeddings. We calculated the difference of the running time (in seconds) as percentage with the following formula:


\begin{equation*}\frac{{{{T}_{\ 1}} - \ {{T}_2}}}{{{{T}_1}}}\ \times \ 100\end{equation*}

Being T1 the running time of Metapath2Vec and T2 the running time of either BioKG2vec or Dlemb. The experiment was conducted on an 8-core intel i7 machine. The experiment was conducted on an 8-core intel i7 machine.

### Intervertebral disc degeneration biomarker prediction

We tested the model to predict genes associated with IDD. We used the model selected through grid search cross validation with concatenation of the embeddings for the GDAs representation. Lastly, we performed a function enrichment analysis using g:Profiler (50) on the set of prioritized genes with a probability >0.95 to be associated to IDD.

## Results

### Data integration and KG structure

We integrated multiple sources of data in the form of KG for a total of 95 952 nodes and 2 183 603 edges. The KG contains four types of nodes: drugs (*n* = 2991), phenotypes (*n* = 28 374), proteins (*n* = 21 019) and functions (*n* = 43 568). These entities are connected by 81 different types of relationships represented as edges. The relationships are obtained through different data sources, 18 282 proteins interacting among each other (87, 1356 edges), 19 409 proteins annotated to 18 813 biological functions (303 404 edges) and 8053 proteins annotated to 13 525 phenotypes (246 006 edges). Moreover, drug information was included: 1551 drugs annotated to 828 phenotypes for a total of 5744 edges and 2887 connected to 2074 proteins they target for a total of 14 491 edges. The degree distribution of the graph follows a scale free law (Supplementary Figure S1) (51).

### Unsupervised clustering of the embeddings reflects the biological classification

**Figure 1.**
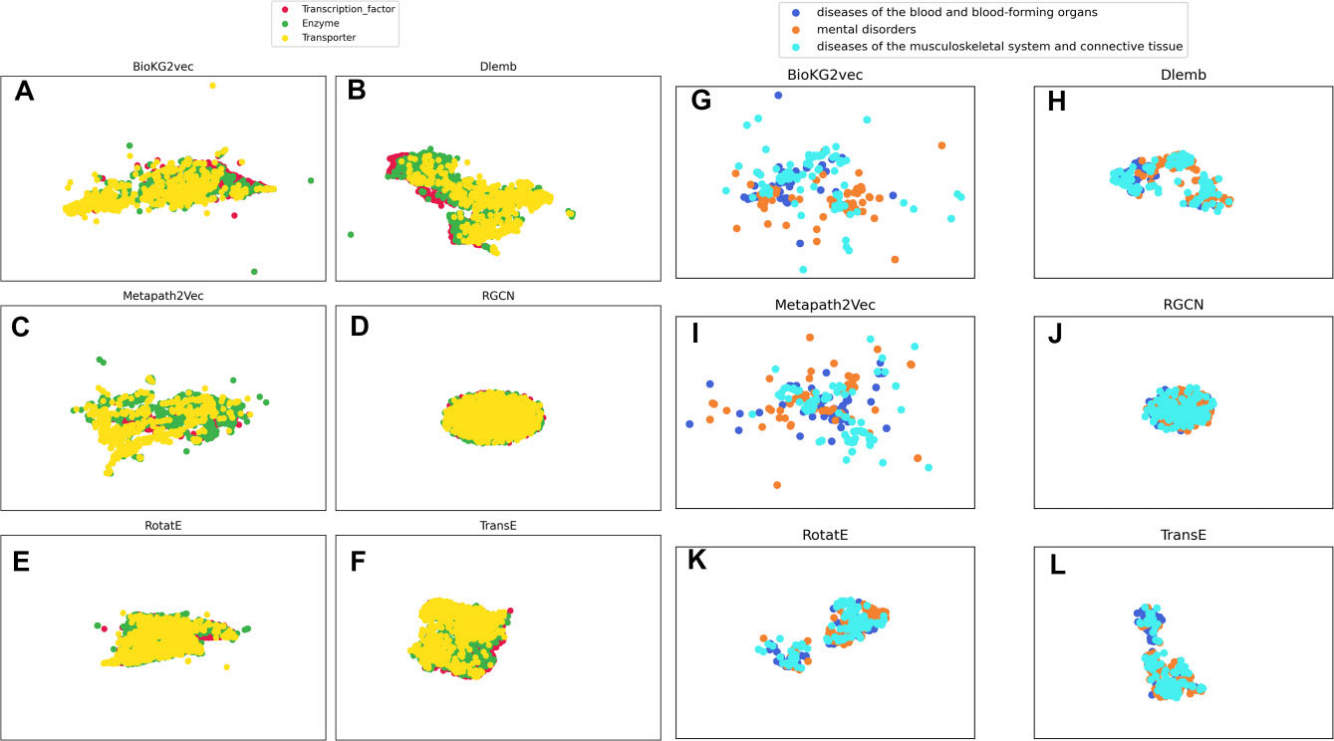
UMAP of gene-embeddings (panels **A–F**) and disease-embeddings (panels **G–L**) generated with BioKG2vec (A, G), Dlemb (B, H), Metapath2Vec (C, I), RGCN (D, J), RotatE (E,K) and TransE (F, L).

From the KG, we generated embeddings using six algorithms. Figure 1 shows the first two UMAP embeddings of genes and diseases. The embeddings tend to differentiate among gene products belonging to different groups: secreted, transcription factors, and transporters (Figure 1A–G). Metapath2Vec, BioKG2vec, and Dlemb from a visual perspective achieve the best clustering of genes. In Figure 1G–L, only three categories of diseases are represented, corresponding to the ICD chapters disease of blood and blood-forming organs, diseases of the musculoskeletal system and connective tissue and mental disorders. As above, algorithms Metapath2Vec, BioKG2vec and Dlemb visually distinguished disease classes better than others.

**Figure 2.**
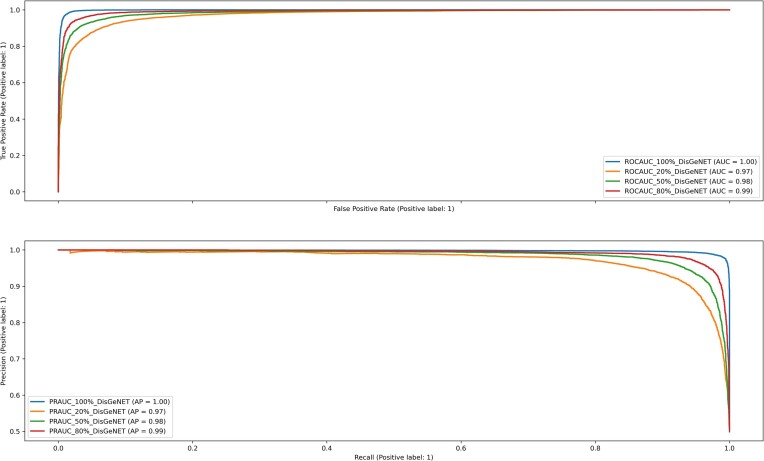
ROCAUC and PRAUC of the prediction of GDAs. Several KG embeddings are obtained using increasing percentages of known GDAs from 20% to 100%. Note: embeddings were generated with Metapath2Vec, using concatenation for combining embeddings and SVM for the classification/prediction algorithm.

The algorithm producing the best clustering of disease classes and gene products was Metapath2Vec, which has a higher homogeneity score for both genes and diseases (Table 2). For the case of diseases, the silhouette score of the embeddings produced with any algorithm couldn’t match the gold standard number of clusters (Supplementary Figures S2 and S3).

**Table 2.** Homogeneity score of K-means algorithm calculated for genes (number of clusters = 16) and diseases (number of clusters = 16). True labels are classification from ICD-9 and HPA for diseases and genes respectively

	Homogeneity score	
	Genes	Diseases
**Metapath2Vec**	**0.49**	**0.28**
**Dlemb**	0.35	0.17
**RotatE**	0.35	0.15
**Trans-E**	0.29	0.09
**BioKG2vec**	0.2	0.20
**RGCN**	0.008	0.02

The embeddings of gene products generated with Metapath2Vec produced more homogeneous clusters. Assigning every different gene and disease correctly to their category is a very complex task because of the high granularity of genes and disease classes (Supplementary Figures S4 and S5).

### Model selection through grid search cross-validation

The best performing combination for GDA prediction was Metapath2Vec. Metapath2Vec coupled with concatenation of the gene and disease embedding as association representation and SVM with parameters *C* = 10 and kernel = rbf as classification algorithm. The whole output of the experiment is available in Supplementary Table S1. The following experiments were run using this combination.

### Heterogeneous data integration and preprocessing

Pre-processing the ontologies leads to better ROCAUC and AUPRC compared to using embeddings generated with raw data. Nevertheless, adding heterogeneous data in the KG did not significantly affect the predictions of GDAs (Table 3). Integrating more data leads to similar performances which can be appreciated when comparing the results of generating the KG using HPO data with HPO and GO data. We must note the different results on the use of Dlemb algorithm (Supplementary Table S2). While the predictive power of Metapath2Vec is not affected by the preprocessing of the ontologies, Dlemb significantly improves the AUPRC and ROCAUC after preprocessing.

**Table 3.** ROCAUC and AUPRC of different experiments of GDAs predictions using Human Phenotype Ontology (HPO) + annotations (A), HPO ontology processed (B) and HPO + Gene Ontology (GO) + GO annotations

Experiment	ROCAUC	AUPRC
HPO + HPO annotations raw	**0.95**	**0.98**
HPO + HPO annotations processed	0.93	0.97
HPO + HPO annotations + GO + GO annotations processed	0.93	0.97

The embeddings were generated with Metapath2Vec and we used SVM as predictive algorithm, and operator concatenation for combining the embeddings.

### The amount of training GDAs in the KG affects the prediction of GDAs

We tested the effect on the predictions caused by the increase of GDAs in the KG. We expect that increasing the amount of GDAs in the KG will increase the quality of the predictions. Figure 2 shows that the increase in the number of GDAs used for training the knowledge graph embeddings increases the values of ROCAUC and AUPRC.

### Comparison with randomly generated embeddings

Supplementary Table S3 presents the outcomes of the experiment contrasting embeddings produced by Metapath2Vec with those generated randomly. Metapath2Vec embeddings reach an average ROCAUC of 0.93 while random generated embeddings have random metrics. These results are due to the biological information intrinsic to the embeddings since the effect of the number of GDAs was prevented by selecting associations of one disease only. In fact, the number of associations is a latent variable that is learned by the model.

### Model generalization across different disease classes

**Figure 3.**
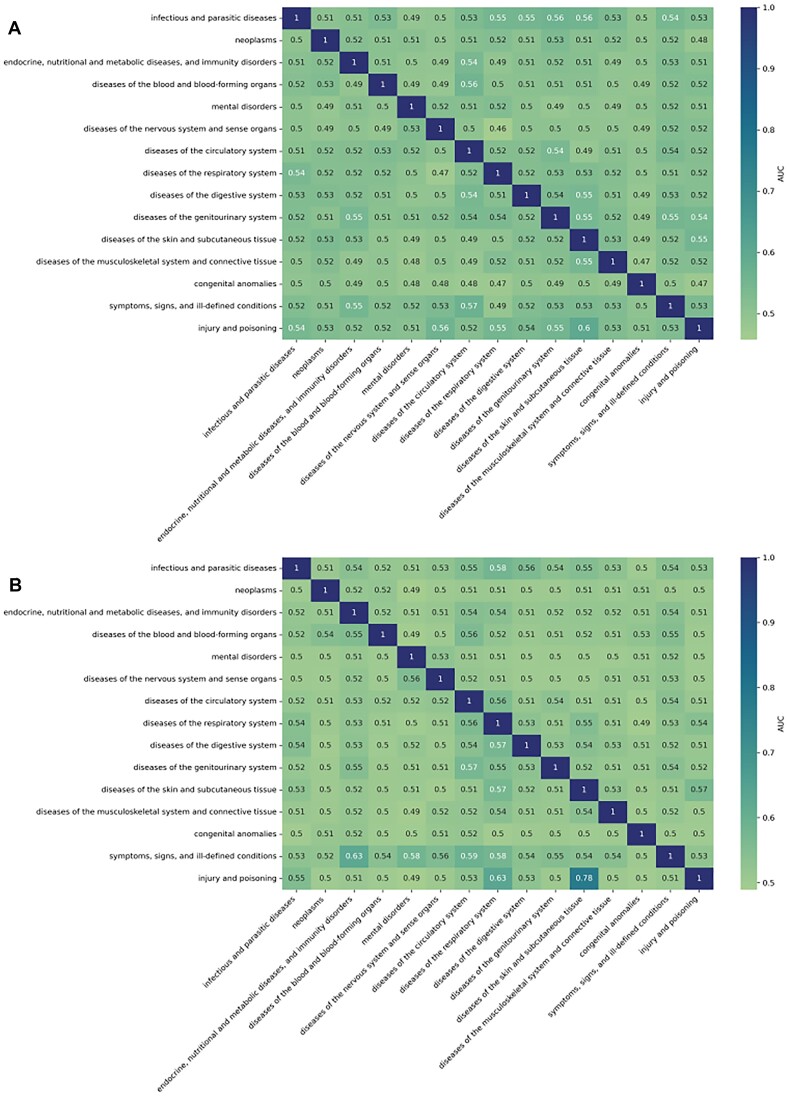
ROCAUC of the best performing combination for the prediction on different disease classes. (**A**) Results for randomly generated embeddings, the ROCAUC shows random values. (**B**) The embeddings were generated with Metapath2Vec, the GDAs representation was concatenation, and the algorithm was SVM with parameters *C* = 10 and kernel = rbf. We show the results of training a model on a specific ICD-9 disease class (rows), and then testing on the others (columns).

Figure 3 shows the performance of the model trained on a specific ICD9 disease class and tested on all the others. Training and testing in diseases belonging to the same class leads to accurate predictions. However, embeddings generated with Metapath2Vec have poor prediction capabilities across different ICD-9 classes. Similar results were observed with randomly generated embeddings. Biological information encoded in Dlemb generated embeddings is translated across disease classes and we can see that some pairs of disease classes achieved a noteworthy prediction (e.g. the model trained for neoplasms predicts genes associated with diseases of circulatory system with an ROCAUC > 0.7) (Supplementary Figure S6). As expected, randomly generated embeddings show ROCAUC in the heatmap with random values.

### Computational performance of the best algorithms for KGE

We compared the performance of the three algorithms that reached the highest ROCAUC during the grid search cross validation in terms of running time. In the Supplementary Figure S7 are reported the running times of 10 experiments. BioKG2vec and Dlemb are respectively ∼100% and 360% faster than Metapath2Vec.

### KGE successfully predict genes associated to IDD

Finally, we used the selected prediction model with the best parametrization to predict GDAs for IDD. IDD is one of the main causes of low back pain, the largest cause of morbidity worldwide affecting 80% of people from Western countries during their lifetime (52). IDD consists of the gradual deterioration of the intervertebral disc (IVD) in which the content of collagen and glycosaminoglycan decreases, and it becomes more dehydrated and fibrotic. Due to this, its anatomical areas nucleus pulposus (NP) and anulus fibrosus (AF) becomes less distinguishable (53). Also, during IDD there is a catabolic shift in the biochemical processes of the disc environment with an increased expression of matrix degrading enzymes promoted by catabolic cytokines and vascularization of the tissues (54). According to DisGeNET (curated sources), IDD is associated to TGFβ-1, HTRA1 and SPARC. We ran predictions for 20951 genes, of those 445 were predicted to be associated to the disease and 93 with a probability > 0.95. The results of the top 10 prioritized genes are shown in Table 4.

**Table 4.** Top 10 genes prioritized from the model with highest predictive capabilities

Gene ID	Gene Symbol	Probability
7040	TGFB1	1
4088	SMAD3	1
4318	MMP9	1
4015	LOX	1
7043	TGFB3	1
7046	TGFBR1	1
7042	TGFB2	1
1277	COL1A1	1
4313	MMP2	1
4087	SMAD2	1

The predictive analysis identifies the TGFβ-1 gene as the most promising candidate associated with Intervertebral Disc Degeneration (IDD), with isoforms TGFβ-2 and TGFβ-3 also receiving prioritization. Notably, TGFβ-1 emerges as the highest-scoring gene in DisGeNET’s curated dataset related to disc degeneration. TGFβ plays a multifaceted role in various pathways associated with the homeostasis and turnover of the extracellular matrix in IDD (55). Additionally, SMAD3 and SMAD2, integral genes in disc homeostasis, participate in the TGF-β pathway. (56). Matrix metalloproteinase 9 (MMP9) and matrix metalloproteinase 2 (MMP2) enzymes contribute significantly to IDD by participating in matrix degradation, targeting proteins expressed in the intervertebral disc like collagens and aggrecan. (57). Moreover, LOX, crucial for cartilage homeostasis, presents a potential strategy for cartilage regeneration(58), with studies indicating its anti-apoptotic effects in TNF-α treated rat NP-cells. (59). These genes were shown to have a role in IDD and could be further investigated to elucidate the mechanisms that lead to the degeneration of the disc.

**Figure 4.**
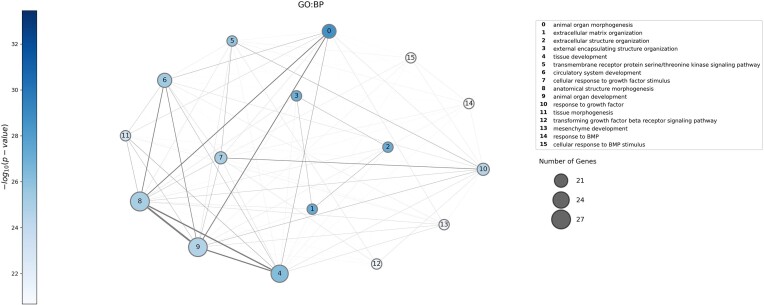
Gene ontology biological processes (GO:BP) function enrichment analysis on the genes with probability higher than 0.95 to be associated to C0158266 (*n* = 93). To run the functional enrichment, we used g:Profiler. The nodes correspond to the pathway enriched in the gene set, their size is proportional to the number of genes belonging to that specific pathway and the colour is related to the significance of the enrichment in the gene set (calculated through hypergeometric distribution). An edge exists between two nodes if there are genes shared between the two pathways and the width of the edge is proportional to the number of the genes shared.

To further explore the biological functions of these candidate genes, we performed a function enrichment analysis (Figure 4). The top prioritized genes are enriched in processes related to the extracellular matrix organization, pathways related to collagen formation, and extracellular matrix degradation, all of them related to IDD.

## Discussion

In this work, we investigated how KGE perform to predict gene–disease associations. First, we generated a KG by implementing heterogeneous biological information such as protein–protein interactions, gene–disease associations, drug–disease associations and drug–protein interactions and ontologies. The integration of multiple knowledge-based datasets prevented us from using syntactic-based approaches for embedding-creation such as OPA2VEC (60). Syntactic approaches rely in the set of axioms only for obtaining the embeddings without the intermediate graph-based representation (61), so the input of the algorithm must be in Web-Ontology Language (OWL) format. Moreover, the integration of different ontologies is a challenging task and an active research topic (62).

In this study, we systematically assessed diverse methodologies for KGE construction and introduced two novel algorithms, namely BioKG2vec and Dlemb. Our comprehensive evaluation reveals that these algorithms exhibit superior performance compared to most existing methods. Notably, the parallelized implementation of both BioKG2vec and Dlemb results in substantially reduced running times in comparison to Metapath2Vec. This enhanced scalability facilitates the effective utilization of computational resources.

We conducted an extensive analysis of embeddings utilizing unsupervised machine learning techniques. Our investigation encompassed the integration of diverse data types and the comparison of GDA predictions using random features. Our findings revealed that augmenting the proportion of GDA within the KG enhances model performance. This observation suggests that task-specific embeddings implementation could enhance predictions, potentially leveraging the learning of pertinent features, as indicated elsewhere (17). Furthermore, we applied KGE to prioritize new genes associated with IDD, illustrating their utility in inferring disease biomarkers even in scenarios with limited genetic data. Notably, our model, trained on a DisGeNET curated dataset containing merely three associations, prioritized 445 genes, which effectively reflected the underlying biology of IDD. In fact, the polygenic nature and epistatic interactions characteristic of non-communicable diseases pose challenges to comprehending the intricate biology underlying the development of complex conditions (63).

Finally, we emphasize the significance of scrutinizing the data quality employed in embedding creation, as predictive models can glean numerous latent features, potentially introducing bias to the outcomes.

## Conclusions

In this work, we carried out an extensive investigation on KGE from the generation and evaluation of the produced embeddings to the development of two new models for KGE generation and the utilization of the created embedding in a GDA prediction task. We showed that embeddings can effectively be implemented in the biomedical field to infer new knowledge over a certain domain. Nevertheless, many challenges remain open that require interdisciplinary collaboration to reach better outcomes in the healthcare sector.

## Supplementary Material

lqae049_Supplemental_File

## Data Availability

The code to reproduce the experiment of the paper is available at: https://zenodo.org/doi/10.5281/zenodo.11048450. The tool for Biomarker prediction with the implementation of the top 3 best performing algorithms (Metapath2Vec, Dlemb and BioKG2vec) is available at: https://zenodo.org/doi/10.5281/zenodo.11048634. The implementation of BioKG2vec is available at: https://zenodo.org/doi/10.5281/zenodo.8233717. The implementation of Dlemb is available at: https://zenodo.org/doi/10.5281/zenodo.8233703.
