# Supplementary material for: Predicting gene disease associations with knowledge graph embeddings for diseases with curtailed information
Source: NAR Genom Bioinform. 2024 May 14;6(2):lqae049. doi: 10.1093/nargab/lqae049 (PMC11091931; doi:10.1093/nargab/lqae049)
Supplement: lqae049_Supplemental_File [file lqae049_supplemental_file.docx]

SUPPLEMENTARY MATERIAL


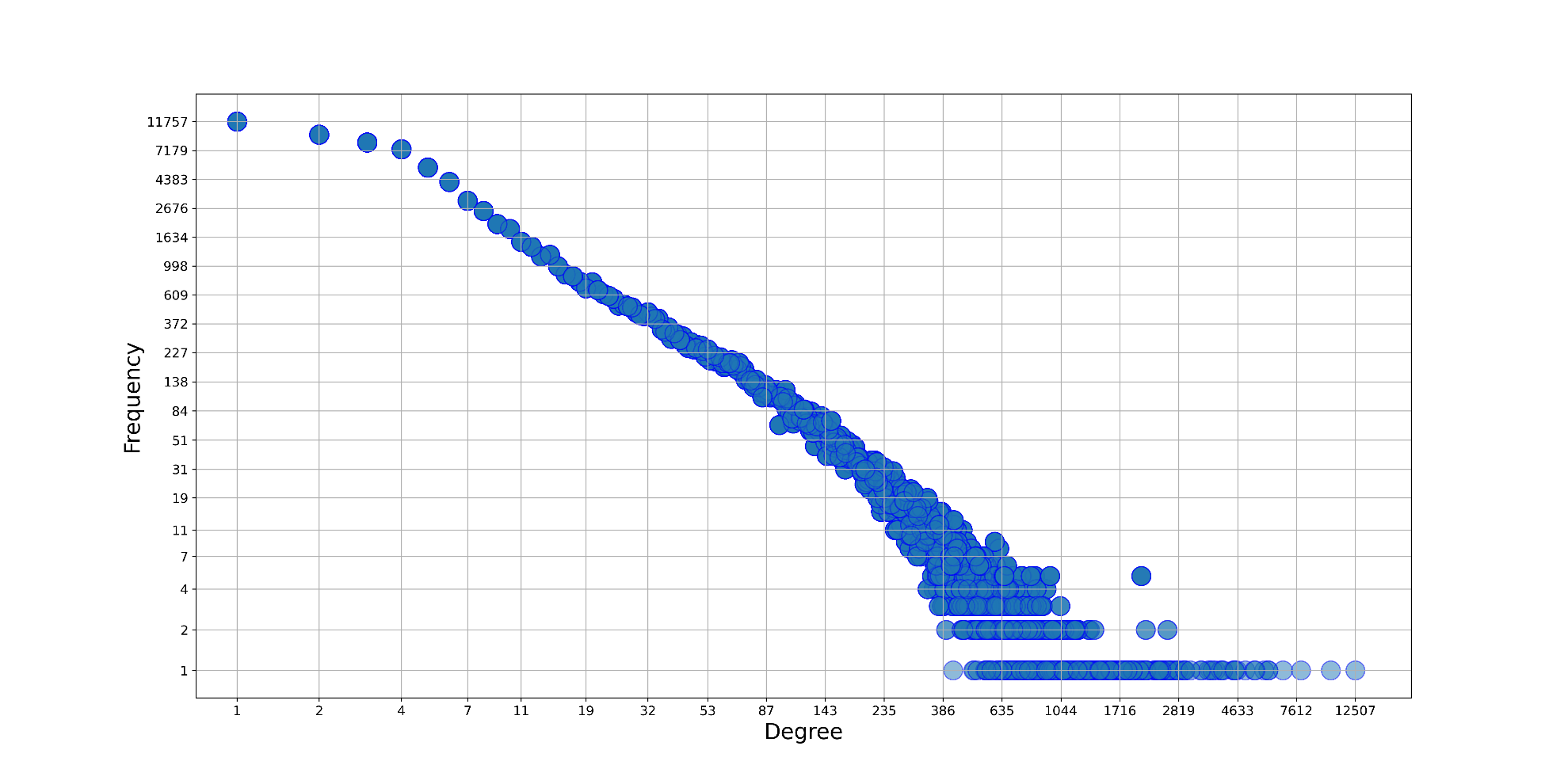


*Supplementary Figure 1: Degree distribution of the knowledge graph, the x-axis represents the degree i.e. number of edges adjacent to a specific node and the y-axis is the frequency of nodes with that specific degree in the graph. The nodes in the KG follows a scale free degree distribution.*

*
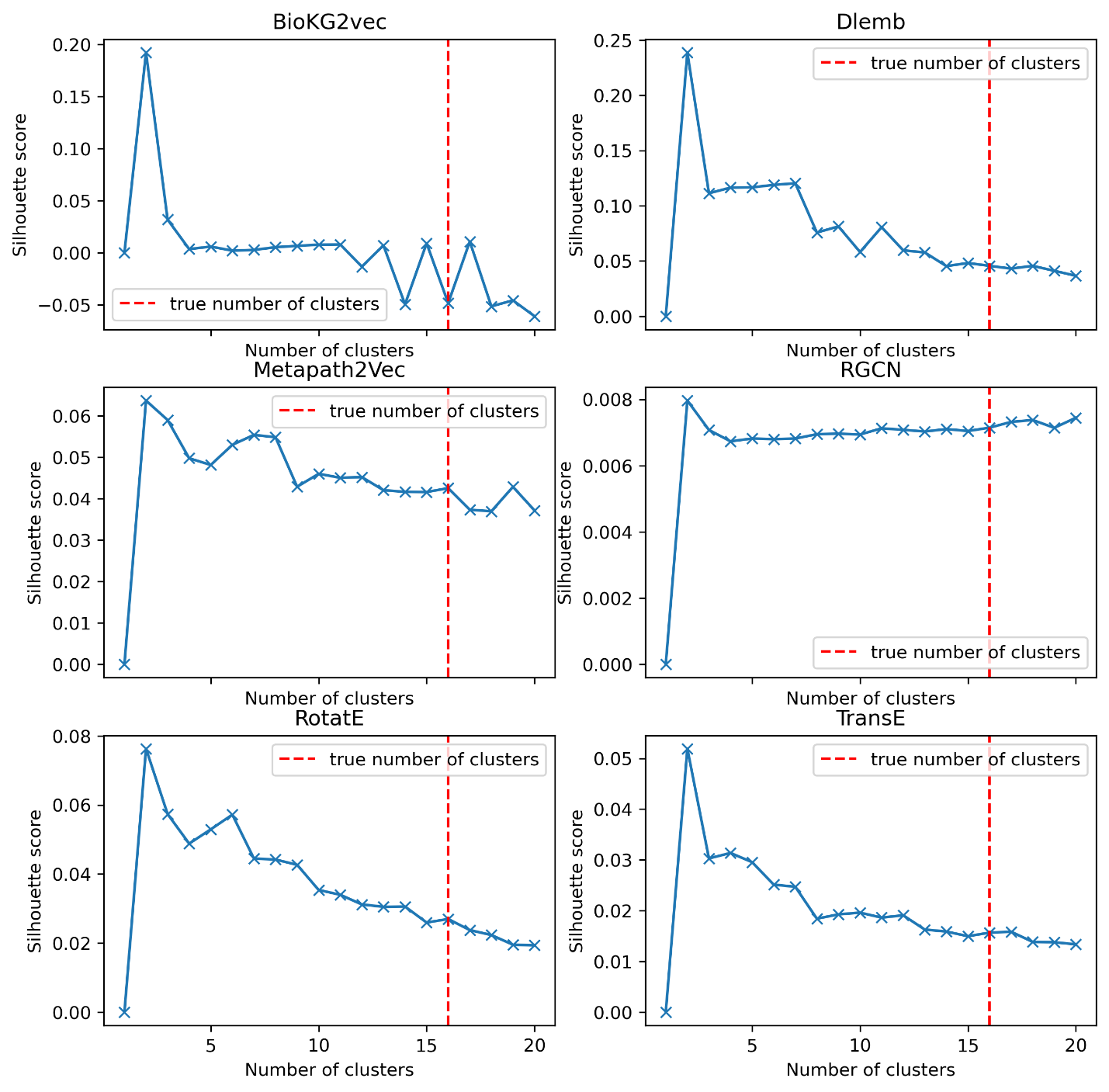
*

*Supplementary Figure 2: Silhouette scores calculated for different numbers of K-means clusters for gene embeddings. The red line represents n = 16 i.e. the actual number of gene classes from Human Protein Atlas.*

*
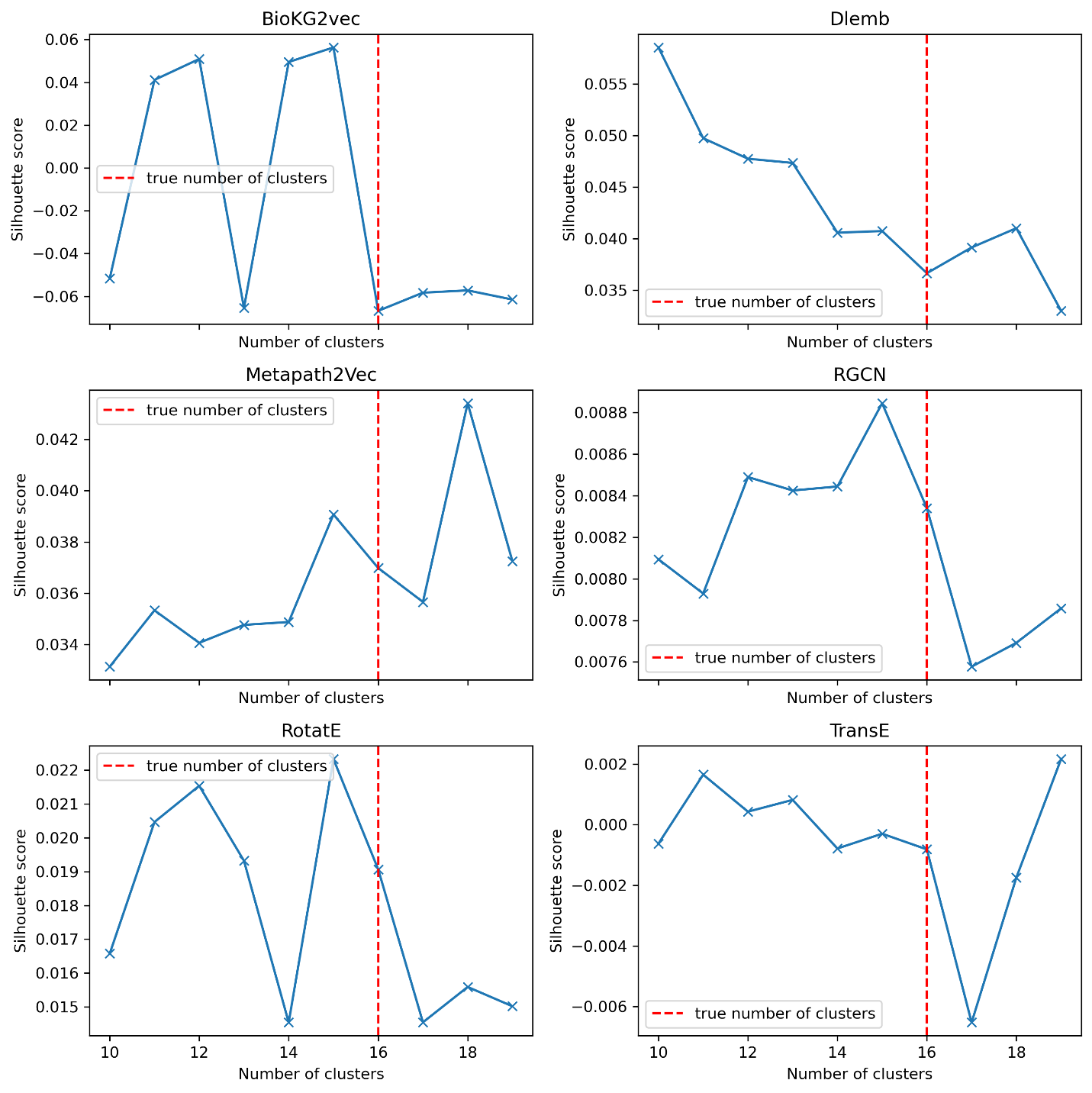
*

*Supplementary Figure 3: Silhouette score calculated for different numbers of K-means clusters of diseases embeddings. The red line represents n = 16 i.e. the actual number of disease classes from ICD-9.*


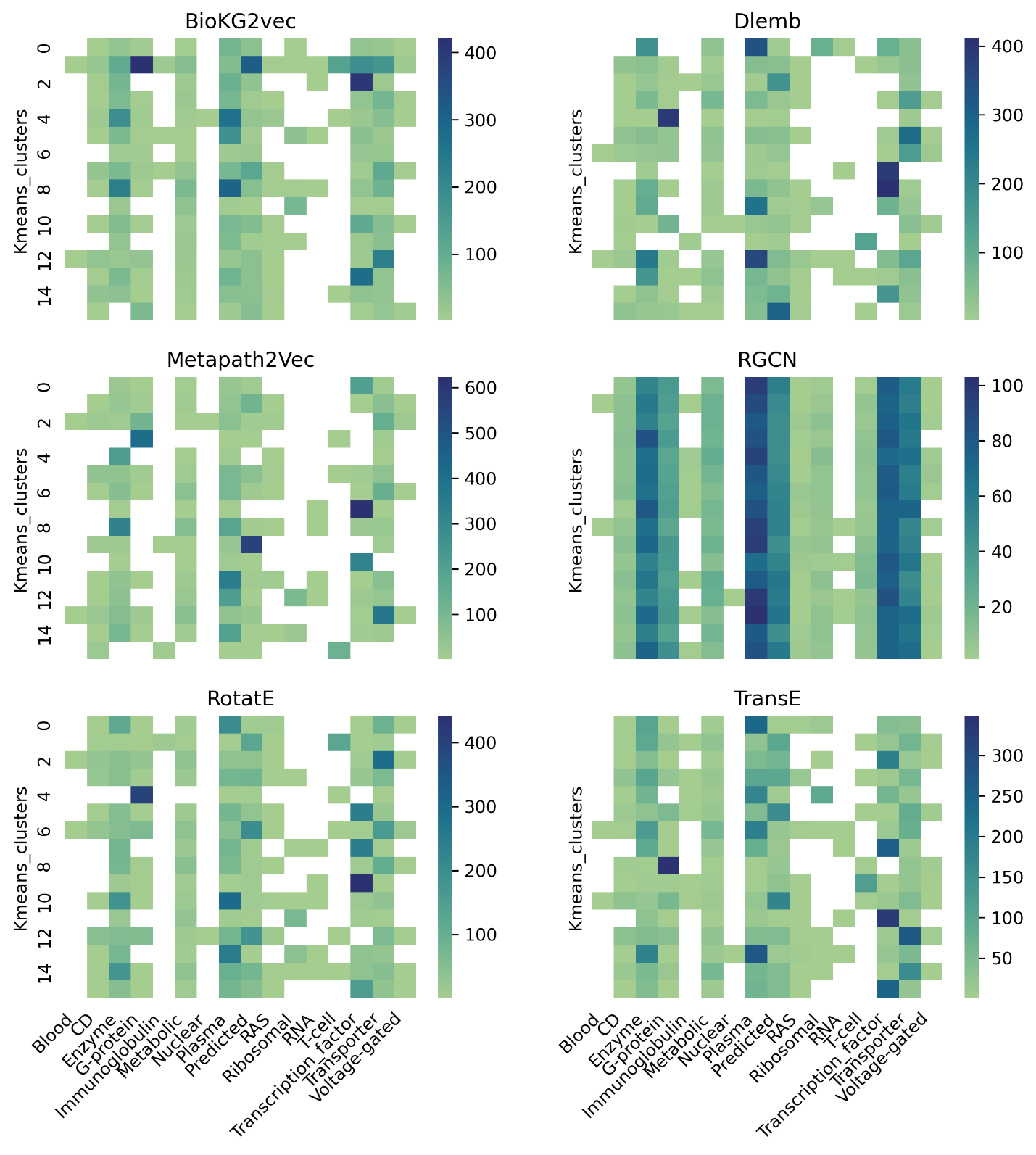


*Supplementary Figure 4: K-means clusters on gene product embeddings separated by Human Protein Atlas protein categories. On the y axis are the 16 clusters produced from the algorithm and on the x axis the protein classes. The color indicates the number of gene products in each cluster.*


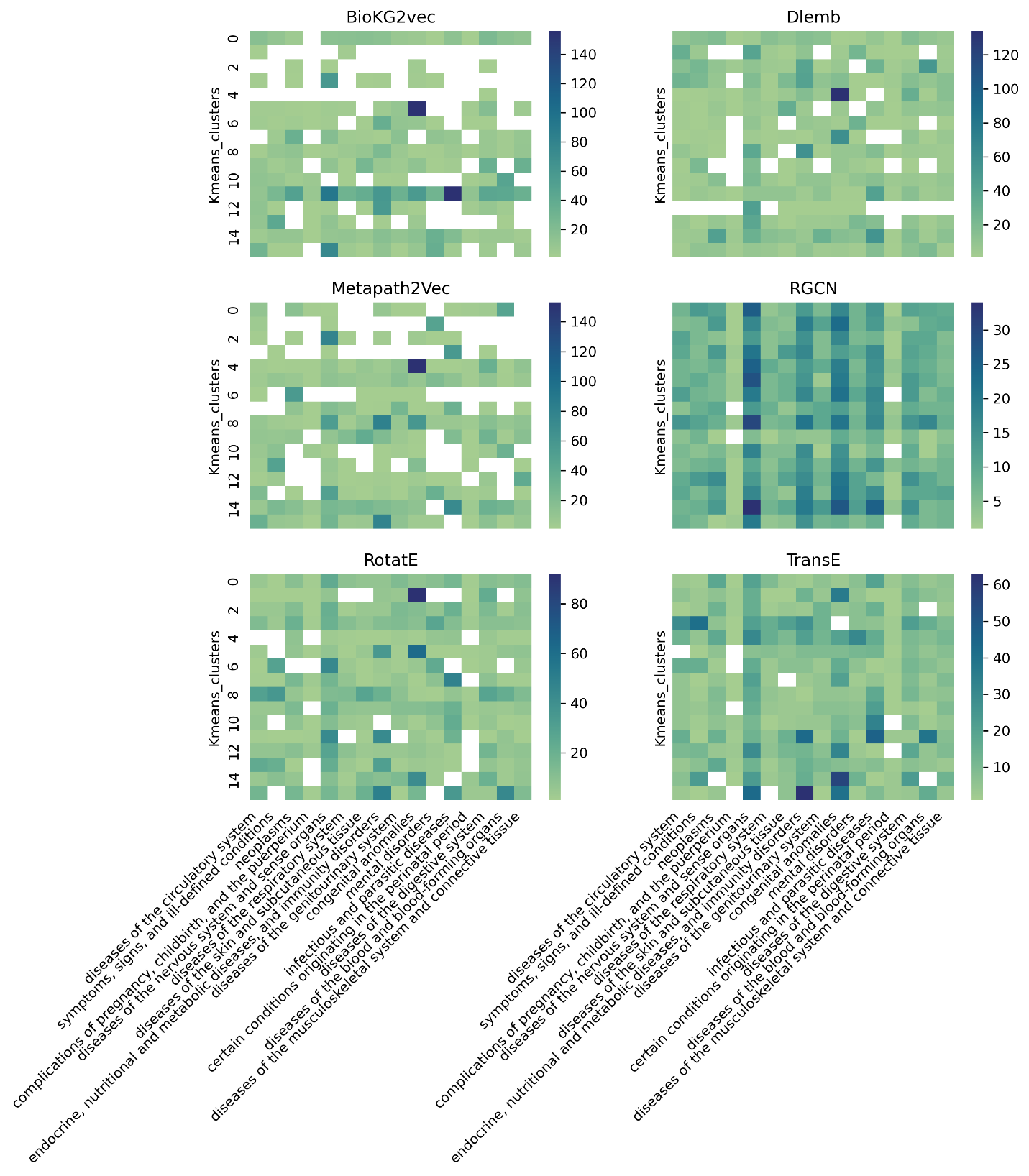


*Supplementary Figure 5: K-means clusters on disease embeddings separated by ICD-9 disease codes. On the y axis are the 16 clusters produced from the algorithm and on the x axis the disease classes. The color indicates the number of diseases in each cluster. BioKG2vec*

Supplementary Table 1: Results of grid search cross-validation. The results are ordered by ROCAUC. DLemb Metapath2vec and BioKG2vec with Concatenation GDA representations are the best performing algorithms. Svm is support vector machine, lr logistic regression, xgb xgboost, rf random forest, and ffn feedforward neural network.

| **Combination** | **F1** | **PRECISION** | **RECALL** | **ACCURACY** | **ROCAUC** | **PRAUC** |
| --- | --- | --- | --- | --- | --- | --- |
| **Metapath2vec_Concatenation_svm** | 0.88 | 0.97 | 0.79 | 0.88 | 0.88 | 0.87 |
| **Metapath2vec_Sum_svm** | 0.89 | 0.94 | 0.83 | 0.89 | 0.89 | 0.86 |
| **BioKG2vec_Concatenation_svm** | 0.88 | 0.94 | 0.8 | 0.88 | 0.88 | 0.85 |
| **Metapath2vec_Concatenation_ffn** | 0.87 | 0.95 | 0.77 | 0.87 | 0.87 | 0.85 |
| **Metapath2vec_Concatenation_xgb** | 0.87 | 0.94 | 0.79 | 0.87 | 0.87 | 0.85 |
| **BioKG2vec_Concatenation_ffn** | 0.88 | 0.92 | 0.83 | 0.88 | 0.88 | 0.84 |
| **Metapath2vec_Average_svm** | 0.86 | 0.95 | 0.75 | 0.86 | 0.86 | 0.84 |
| **Metapath2vec_Average_ffn** | 0.86 | 0.93 | 0.77 | 0.86 | 0.86 | 0.83 |
| **Metapath2vec_Sum_ffn** | 0.86 | 0.92 | 0.79 | 0.86 | 0.86 | 0.83 |
| **DLemb_Concatenation_svm** | 0.87 | 0.89 | 0.84 | 0.87 | 0.87 | 0.83 |
| **DLemb_Concatenation_xgb** | 0.87 | 0.9 | 0.83 | 0.87 | 0.87 | 0.83 |
| **BioKG2vec_Average_svm** | 0.86 | 0.91 | 0.8 | 0.86 | 0.86 | 0.82 |
| **BioKG2vec_Concatenation_xgb** | 0.86 | 0.9 | 0.81 | 0.86 | 0.86 | 0.82 |
| **BioKG2vec_Sum_svm** | 0.86 | 0.87 | 0.85 | 0.86 | 0.86 | 0.81 |
| **RotatE_Concatenation_svm** | 0.85 | 0.89 | 0.79 | 0.85 | 0.85 | 0.81 |
| **DLemb_Concatenation_rf** | 0.85 | 0.88 | 0.82 | 0.85 | 0.85 | 0.81 |
| **Metapath2vec_Hadmard_svm** | 0.82 | 0.94 | 0.7 | 0.83 | 0.83 | 0.8 |
| **BioKG2vec_Average_ffn** | 0.84 | 0.89 | 0.78 | 0.84 | 0.84 | 0.8 |
| **DLemb_Hadmard_xgb** | 0.84 | 0.88 | 0.79 | 0.84 | 0.84 | 0.8 |
| **BioKG2vec_Sum_ffn** | 0.84 | 0.89 | 0.77 | 0.84 | 0.84 | 0.8 |
| **DLemb_Hadmard_rf** | 0.84 | 0.88 | 0.77 | 0.84 | 0.84 | 0.8 |
| **Metapath2vec_Hadmard_xgb** | 0.82 | 0.93 | 0.7 | 0.82 | 0.82 | 0.8 |
| **Metapath2vec_Sum_xgb** | 0.83 | 0.89 | 0.75 | 0.83 | 0.83 | 0.79 |
| **Metapath2vec_Average_xgb** | 0.83 | 0.89 | 0.75 | 0.83 | 0.83 | 0.79 |
| **DLemb_Concatenation_ffn** | 0.84 | 0.85 | 0.83 | 0.84 | 0.84 | 0.79 |
| **Metapath2vec_Hadmard_ffn** | 0.81 | 0.93 | 0.68 | 0.82 | 0.81 | 0.79 |
| **Metapath2vec_Concatenation_rf** | 0.83 | 0.87 | 0.78 | 0.83 | 0.83 | 0.79 |
| **DLemb_Sum_svm** | 0.83 | 0.87 | 0.78 | 0.84 | 0.83 | 0.79 |
| **RotatE_Sum_svm** | 0.83 | 0.88 | 0.77 | 0.83 | 0.83 | 0.79 |
| **DLemb_Average_svm** | 0.83 | 0.87 | 0.78 | 0.83 | 0.83 | 0.78 |
| **DLemb_Hadmard_ffn** | 0.83 | 0.87 | 0.77 | 0.83 | 0.83 | 0.78 |
| **DLemb_Hadmard_lr** | 0.83 | 0.86 | 0.77 | 0.83 | 0.83 | 0.78 |
| **DLemb_Hadmard_svm** | 0.83 | 0.86 | 0.78 | 0.83 | 0.83 | 0.78 |
| **BioKG2vec_Hadmard_ffn** | 0.82 | 0.87 | 0.75 | 0.82 | 0.82 | 0.78 |
| **Metapath2vec_Hadmard_lr** | 0.8 | 0.91 | 0.67 | 0.81 | 0.81 | 0.78 |
| **BioKG2vec_Hadmard_svm** | 0.81 | 0.88 | 0.72 | 0.81 | 0.81 | 0.78 |
| **DLemb_Average_rf** | 0.83 | 0.85 | 0.8 | 0.83 | 0.83 | 0.78 |
| **DLemb_Sum_rf** | 0.83 | 0.85 | 0.8 | 0.83 | 0.83 | 0.78 |
| **DLemb_Average_xgb** | 0.82 | 0.86 | 0.78 | 0.82 | 0.82 | 0.78 |
| **DLemb_Sum_xgb** | 0.82 | 0.86 | 0.78 | 0.82 | 0.82 | 0.78 |
| **RotatE_Average_svm** | 0.82 | 0.87 | 0.75 | 0.82 | 0.82 | 0.77 |
| **RotatE_Concatenation_xgb** | 0.82 | 0.86 | 0.77 | 0.82 | 0.82 | 0.77 |
| **Metapath2vec_Hadmard_rf** | 0.8 | 0.89 | 0.69 | 0.8 | 0.8 | 0.77 |
| **BioKG2vec_Hadmard_xgb** | 0.81 | 0.86 | 0.74 | 0.81 | 0.81 | 0.77 |
| **BioKG2vec_Concatenation_rf** | 0.82 | 0.84 | 0.8 | 0.82 | 0.82 | 0.77 |
| **BioKG2vec_Average_xgb** | 0.82 | 0.85 | 0.77 | 0.82 | 0.82 | 0.77 |
| **BioKG2vec_Sum_xgb** | 0.82 | 0.85 | 0.77 | 0.82 | 0.82 | 0.77 |
| **RotatE_Concatenation_ffn** | 0.82 | 0.83 | 0.8 | 0.82 | 0.82 | 0.76 |
| **RotatE_Concatenation_rf** | 0.81 | 0.85 | 0.75 | 0.81 | 0.81 | 0.76 |
| **Metapath2vec_Average_rf** | 0.81 | 0.83 | 0.79 | 0.81 | 0.81 | 0.76 |
| **Metapath2vec_Sum_rf** | 0.81 | 0.83 | 0.79 | 0.81 | 0.81 | 0.76 |
| **RotatE_Sum_ffn** | 0.8 | 0.83 | 0.75 | 0.8 | 0.8 | 0.75 |
| **BioKG2vec_Hadmard_lr** | 0.78 | 0.86 | 0.68 | 0.79 | 0.79 | 0.74 |
| **TransE_Concatenation_svm** | 0.8 | 0.82 | 0.75 | 0.8 | 0.8 | 0.74 |
| **RotatE_Average_xgb** | 0.79 | 0.84 | 0.71 | 0.79 | 0.79 | 0.74 |
| **RotatE_Sum_xgb** | 0.79 | 0.84 | 0.71 | 0.79 | 0.79 | 0.74 |
| **DLemb_Sum_ffn** | 0.79 | 0.8 | 0.78 | 0.8 | 0.79 | 0.73 |
| **BioKG2vec_Sum_rf** | 0.8 | 0.79 | 0.8 | 0.8 | 0.8 | 0.73 |
| **RotatE_Sum_rf** | 0.78 | 0.84 | 0.69 | 0.78 | 0.78 | 0.73 |
| **RGCN_Concatenation_svm** | 0.78 | 0.84 | 0.69 | 0.78 | 0.78 | 0.73 |
| **RotatE_Average_rf** | 0.78 | 0.83 | 0.69 | 0.78 | 0.78 | 0.73 |
| **BioKG2vec_Hadmard_rf** | 0.79 | 0.8 | 0.78 | 0.79 | 0.79 | 0.73 |
| **BioKG2vec_Average_rf** | 0.79 | 0.79 | 0.8 | 0.79 | 0.79 | 0.73 |
| **RotatE_Hadmard_xgb** | 0.77 | 0.84 | 0.68 | 0.77 | 0.77 | 0.73 |
| **RotatE_Hadmard_rf** | 0.77 | 0.83 | 0.68 | 0.77 | 0.77 | 0.73 |
| **TransE_Concatenation_rf** | 0.78 | 0.81 | 0.72 | 0.78 | 0.78 | 0.72 |
| **DLemb_Average_ffn** | 0.78 | 0.79 | 0.77 | 0.78 | 0.78 | 0.72 |
| **RotatE_Hadmard_svm** | 0.77 | 0.83 | 0.68 | 0.77 | 0.77 | 0.72 |
| **TransE_Concatenation_xgb** | 0.78 | 0.8 | 0.75 | 0.78 | 0.78 | 0.72 |
| **RotatE_Average_ffn** | 0.78 | 0.78 | 0.77 | 0.78 | 0.78 | 0.72 |
| **RGCN_Average_svm** | 0.75 | 0.83 | 0.63 | 0.75 | 0.75 | 0.71 |
| **RGCN_Concatenation_xgb** | 0.75 | 0.82 | 0.65 | 0.75 | 0.75 | 0.7 |
| **RotatE_Hadmard_ffn** | 0.75 | 0.8 | 0.68 | 0.76 | 0.76 | 0.7 |
| **RotatE_Hadmard_lr** | 0.74 | 0.81 | 0.64 | 0.75 | 0.75 | 0.7 |
| **RGCN_Concatenation_rf** | 0.75 | 0.79 | 0.67 | 0.75 | 0.75 | 0.69 |
| **TransE_Concatenation_ffn** | 0.75 | 0.76 | 0.74 | 0.75 | 0.75 | 0.69 |
| **DLemb_Concatenation_lr** | 0.75 | 0.76 | 0.72 | 0.75 | 0.75 | 0.69 |
| **TransE_Sum_svm** | 0.74 | 0.76 | 0.7 | 0.74 | 0.74 | 0.68 |
| **RotatE_Concatenation_lr** | 0.74 | 0.75 | 0.72 | 0.74 | 0.74 | 0.68 |
| **Metapath2vec_Concatenation_lr** | 0.73 | 0.75 | 0.7 | 0.73 | 0.73 | 0.67 |
| **RGCN_Sum_svm** | 0.73 | 0.74 | 0.7 | 0.73 | 0.73 | 0.67 |
| **BioKG2vec_Concatenation_lr** | 0.73 | 0.74 | 0.7 | 0.73 | 0.73 | 0.67 |
| **TransE_Concatenation_lr** | 0.73 | 0.74 | 0.71 | 0.73 | 0.73 | 0.67 |
| **TransE_Average_svm** | 0.71 | 0.74 | 0.65 | 0.71 | 0.71 | 0.66 |
| **TransE_Hadmard_xgb** | 0.71 | 0.74 | 0.65 | 0.71 | 0.71 | 0.66 |
| **Metapath2vec_Sum_lr** | 0.72 | 0.73 | 0.69 | 0.72 | 0.72 | 0.66 |
| **Metapath2vec_Average_lr** | 0.72 | 0.73 | 0.69 | 0.72 | 0.72 | 0.66 |
| **TransE_Hadmard_rf** | 0.71 | 0.75 | 0.63 | 0.71 | 0.71 | 0.65 |
| **BioKG2vec_Sum_lr** | 0.71 | 0.72 | 0.69 | 0.71 | 0.71 | 0.65 |
| **BioKG2vec_Average_lr** | 0.71 | 0.72 | 0.69 | 0.71 | 0.71 | 0.65 |
| **DLemb_Average_lr** | 0.71 | 0.71 | 0.7 | 0.71 | 0.71 | 0.65 |
| **DLemb_Sum_lr** | 0.71 | 0.71 | 0.7 | 0.71 | 0.71 | 0.65 |
| **TransE_Average_rf** | 0.71 | 0.73 | 0.66 | 0.71 | 0.71 | 0.65 |
| **TransE_Sum_rf** | 0.7 | 0.73 | 0.65 | 0.71 | 0.71 | 0.65 |
| **TransE_Sum_xgb** | 0.71 | 0.72 | 0.68 | 0.71 | 0.71 | 0.65 |
| **TransE_Average_xgb** | 0.71 | 0.72 | 0.68 | 0.71 | 0.71 | 0.65 |
| **RGCN_Concatenation_ffn** | 0.7 | 0.72 | 0.65 | 0.7 | 0.7 | 0.65 |
| **TransE_Sum_ffn** | 0.71 | 0.71 | 0.7 | 0.71 | 0.71 | 0.65 |
| **RotatE_Sum_lr** | 0.7 | 0.71 | 0.69 | 0.7 | 0.7 | 0.64 |
| **RotatE_Average_lr** | 0.7 | 0.71 | 0.69 | 0.7 | 0.7 | 0.64 |
| **TransE_Hadmard_svm** | 0.69 | 0.72 | 0.62 | 0.69 | 0.69 | 0.64 |
| **TransE_Hadmard_lr** | 0.69 | 0.72 | 0.62 | 0.69 | 0.69 | 0.63 |
| **TransE_Hadmard_ffn** | 0.69 | 0.7 | 0.65 | 0.69 | 0.69 | 0.63 |
| **TransE_Average_ffn** | 0.68 | 0.71 | 0.6 | 0.68 | 0.68 | 0.63 |
| **TransE_Sum_lr** | 0.67 | 0.66 | 0.68 | 0.67 | 0.67 | 0.61 |
| **TransE_Average_lr** | 0.67 | 0.66 | 0.67 | 0.67 | 0.67 | 0.61 |
| **RGCN_Average_ffn** | 0.65 | 0.67 | 0.59 | 0.65 | 0.65 | 0.6 |
| **RGCN_Sum_ffn** | 0.65 | 0.65 | 0.64 | 0.65 | 0.65 | 0.6 |
| **RGCN_Average_rf** | 0.6 | 0.6 | 0.61 | 0.6 | 0.6 | 0.56 |
| **RGCN_Sum_rf** | 0.59 | 0.59 | 0.59 | 0.59 | 0.59 | 0.55 |
| **RGCN_Average_xgb** | 0.58 | 0.58 | 0.6 | 0.58 | 0.58 | 0.55 |
| **RGCN_Sum_xgb** | 0.58 | 0.58 | 0.6 | 0.58 | 0.58 | 0.55 |
| **RGCN_Concatenation_lr** | 0.56 | 0.56 | 0.59 | 0.56 | 0.56 | 0.53 |
| **RGCN_Hadmard_xgb** | 0.54 | 0.53 | 0.63 | 0.54 | 0.54 | 0.52 |
| **RGCN_Average_lr** | 0.54 | 0.54 | 0.59 | 0.54 | 0.54 | 0.52 |
| **RGCN_Sum_lr** | 0.54 | 0.54 | 0.59 | 0.54 | 0.54 | 0.52 |
| **RGCN_Hadmard_rf** | 0.48 | 0.51 | 0.81 | 0.53 | 0.53 | 0.51 |
| **RGCN_Hadmard_svm** | 0.51 | 0.51 | 0.64 | 0.51 | 0.51 | 0.5 |
| **RGCN_Hadmard_ffn** | 0.51 | 0.51 | 0.6 | 0.51 | 0.51 | 0.5 |
| **RGCN_Hadmard_lr** | 0.49 | 0.5 | 0.65 | 0.5 | 0.5 | 0.5 |

*Supplementary Table 2 effect of data preprocessing and integration on ROCAUC and PRAUC using DLemb algorithm. Preprocessing of the data significantly improves predictive performance of the model.*

| Experiment | ROCAUC | AUPRC |
| --- | --- | --- |
| HPO + HPO annotations raw | 0.76 | 0.87 |
| HPO + HPO annotations processed | **0.92** | **0.96** |
| HPO + HPO annotations + GO + GO annotations processed | 0.92 | 0.93 |

*Supplementary Table 3: Results of 5-fold cross-validation on randomly selected diseases belonging to different number of GDA stratifications (Embedding creation algorithm is Metapath2vec, classification algorithm is support vector machine and GDA representation is concatenation).*

| **model** |  | **Metapath2vec** | | | | | **Random** | | | | |
| --- | --- | --- | --- | --- | --- | --- | --- | --- | --- | --- | --- |
| **CUI** | **number of associations** | **test_accuracy** | **test_precision** | **test_recall** | **test_f1** | **test_roc_auc** | **test_accuracy** | **test_precision** | **test_recall** | **test_f1** | **test_roc_auc** |
| **C0026764** | 42 | 0.88 | 0.92 | 0.85 | 0.88 | 0.95 | 0.58 | 0.57 | 0.6 | 0.56 | 0.66 |
| **C0020517** | 64 | 0.92 | 0.94 | 0.91 | 0.92 | 0.99 | 0.51 | 0.51 | 0.57 | 0.53 | 0.48 |
| **C0013421** | 86 | 0.93 | 0.93 | 0.95 | 0.94 | 0.98 | 0.48 | 0.48 | 0.51 | 0.49 | 0.46 |
| **C0023890** | 103 | 0.89 | 0.89 | 0.9 | 0.89 | 0.96 | 0.46 | 0.45 | 0.45 | 0.45 | 0.43 |
| **C0007134** | 128 | 0.84 | 0.85 | 0.84 | 0.84 | 0.92 | 0.52 | 0.52 | 0.53 | 0.52 | 0.53 |
| **C0032460** | 144 | 0.78 | 0.79 | 0.76 | 0.77 | 0.84 | 0.45 | 0.45 | 0.49 | 0.47 | 0.44 |
| **C0014175** | 161 | 0.85 | 0.85 | 0.84 | 0.84 | 0.92 | 0.49 | 0.49 | 0.48 | 0.49 | 0.46 |
| **C0151744** | 176 | 0.85 | 0.84 | 0.85 | 0.85 | 0.92 | 0.48 | 0.48 | 0.45 | 0.46 | 0.46 |
| **C0015397** | 212 | 0.95 | 0.94 | 0.96 | 0.95 | 0.98 | 0.45 | 0.45 | 0.46 | 0.46 | 0.41 |
| **C0004352** | 261 | 0.86 | 0.85 | 0.87 | 0.86 | 0.93 | 0.45 | 0.45 | 0.45 | 0.45 | 0.42 |


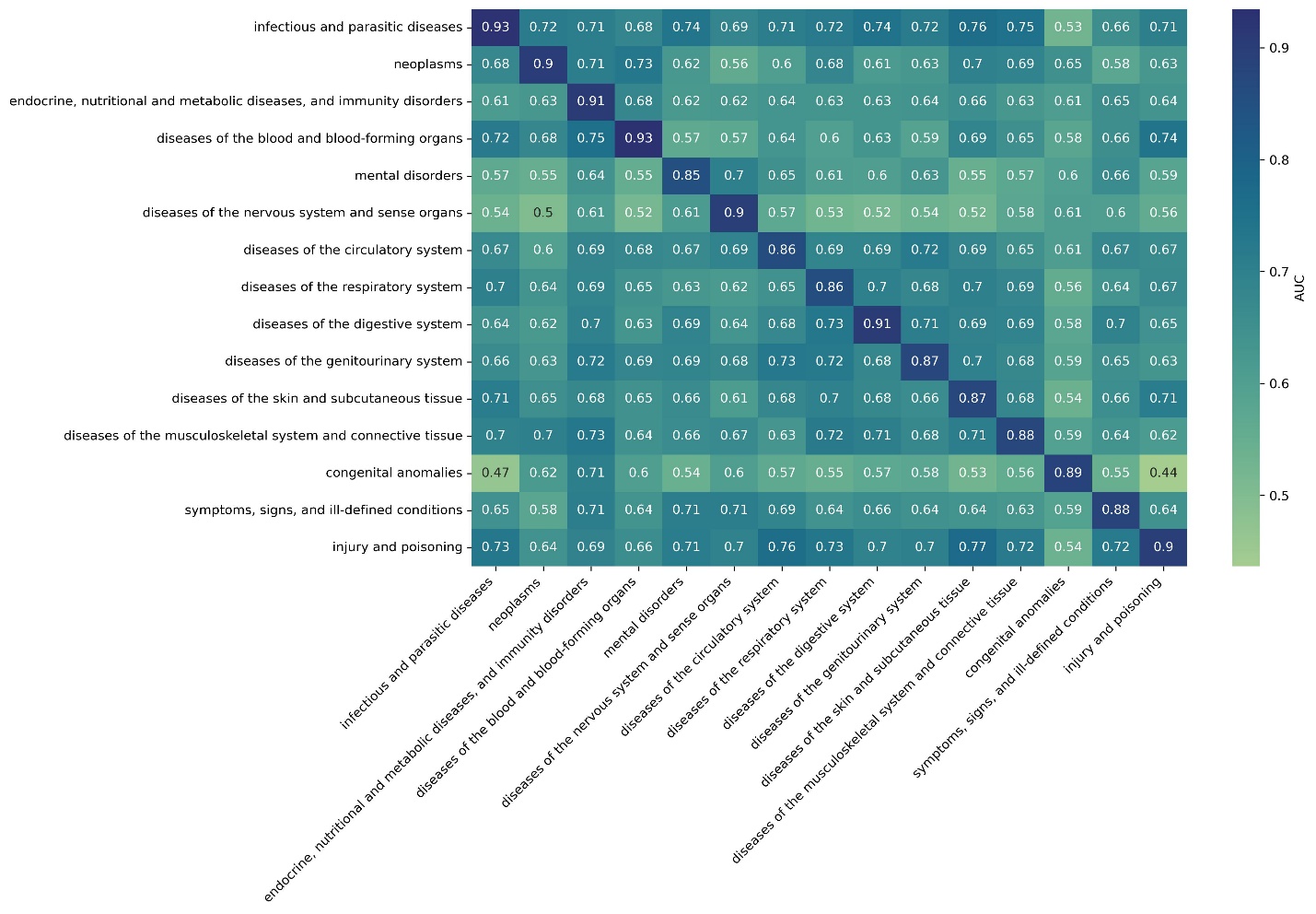


*Supplementary Figure 6: Generalization capabilities of DLemb algorithm, the KGE obtained with the implementation of this algorithm can predict GDAs across different disease classes.*


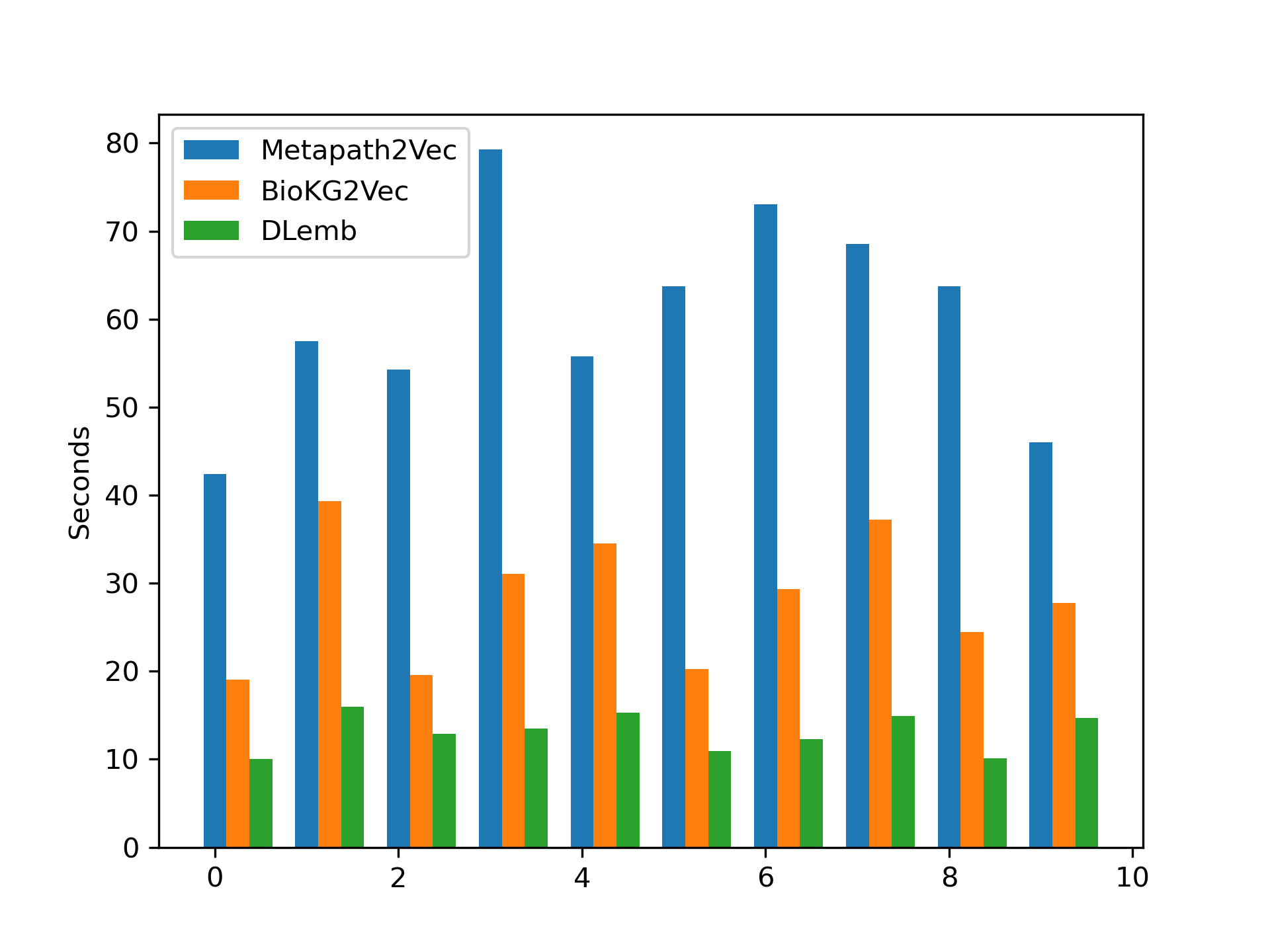


*Supplementary Figure 7: Comparison of algorithms performance. On the x - axis are reported the ten experiments of creating embeddings of subnetworks generated by random sampling 10000 nodes from the knowledge graph, on the y-axis, the time in seconds.*
